# Relationship between risk factors for infertility in women and lead, cadmium, and arsenic blood levels: a cross-sectional study from Taiwan

**DOI:** 10.1186/s12889-015-2564-x

**Published:** 2015-12-09

**Authors:** Hsiao-Ling Lei, Hsiao-Jui Wei, Hsin-Yi Ho, Kai-Wei Liao, Ling-Chu Chien

**Affiliations:** School of Public Health, Taipei Medical University, No. 250, Wuxing St., Taipei City, 110 Taiwan; Infertility Center, Taiwan Adventist Hospital, No. 424, Sec. 2, Bade Rd., Taipei City, 105 Taiwan; Xiamen EMBO Hospital, No. 117-119, Hubin South Road, Xiamen City, 361000 Fujian Province China

**Keywords:** Lead, Cadmium, Arsenic, Female infertility, Chinese herbal medicine

## Abstract

**Background:**

The World Health Organization reported that more than 10 % of women are severely affected by infertility, making the condition a major worldwide public health problem. Lead (Pb), cadmium (Cd), and arsenic (As) are environmental pollutants that may contribute to reproductive disorders. This cross-sectional study investigated the association between blood concentrations of Pb, Cd, and As and risk factors for infertility in women.

**Methods:**

Women who were infertile (*N* = 310) or pregnant (*N* = 57) were recruited from the gynecology and obstetrics department of a hospital. The participants were interviewed to obtain their sociodemographic, reproductive, and lifestyle information. The concentrations of Pb, Cd, and As in their blood samples were measured using inductively coupled plasma mass spectrometry.

**Results:**

Our findings suggested that the concentrations of Pb and As, but not Cd, were significantly higher in the blood of infertile women than in that of pregnant women. A higher percentage of the infertile women consumed more alcohol, used Chinese herbal medicine more frequently, and lacked physical activity compared with the pregnant women. After accounting for potentially relevant predictors, we observed that blood Pb levels might be elevated by using Chinese herbal medicine 1–6 times per week (aOR = 2.82, *p* = 0.05). In addition, engaging in physical activity 1–2 times per week (aOR = 0.37, *p* = 0.05) might assist in reducing Pb accumulation in infertile women, though the p value was borderline.

**Conclusions:**

Lack of physical activity and frequent use of Chinese herbal medicine may be associated with elevated blood Pb levels in infertile women. Chinese herbal medicine use was observed to increase the Pb body burden of both infertile and pregnant women in this study. The risk–benefit for Chinese herbal medicine intake should be evaluated by women of childbearing age.

## Background

The World Health Organization [1] reported that more than 10 % of women are severely affected by infertility. The reproductive health of women of childbearing age is currently a major worldwide public health problem. An increasing number of researchers acknowledge the influence of environmental pollutants, such as heavy metals, organic hydrocarbons, and pesticides from various sources, on public health, particularly in reproductive disorders. Environmental factors, such as exposure to heavy metals, can cause reproductive dysfunction in women [2]; even trace exposure to toxic metals may affect the reproductive health of women [3]. Several studies have illustrated the adverse effects of heavy metals in utero, and the potential reproductive toxicity of these pollutants at levels lower than the tolerable/acceptable daily intake values (ADI) is of serious concern [4–7]. Toxic metals may induce hormonal changes affecting the menstrual cycle, ovulation, and female fertility [8].

Mattison [9] indicated that the impact of environmental pollutants on pregnancy outcomes might have no threshold level, and that the only reasonable approach is to minimize exposures for everyone. Nonessential metals, including lead (Pb), cadmium (Cd), and arsenic (As), are reproductive toxicants widely distributed in the environment [10]. Several epidemiologic studies on menstruation have indicated that metals affect hormone levels [2, 11–14]. Pb and Cd have been identified in human follicular fluid [15–18] and As was shown to cause dose-related increases in ovarian tumors [19]. Randolph et al. [20] reported that women whose infertility could not be otherwise explained may have decreased ovarian sensitivity to gonadotropins, resulting in higher circulating gonadotropin levels, including higher mean serum FSH and LH levels. High levels of serum FSH could indicate poor ovarian function. Krieg [12] examined the associations between blood Pb levels, follicle-stimulating hormone (FSH), and luteinizing hormones (LH). Serum FSH concentrations statistically significantly increased as blood Pb levels increased in women who were postmenopausal, both ovaries removed, and premenopausal (*ß* = 22.2, 32.6, and 8.3, respectively). Chang et al. [21] indicated that women with blood Pb levels higher than 25 μg/L had a 3-fold increased risk of infertility compared with women whose blood Pb levels were less than 25 μg/L.

Although the mechanisms pertaining to the adverse reproductive effects caused by toxic metals have not been fully defined, toxicological studies have provided some insights. Yet, evidence of certain heavy metals contributing to adverse effects on fertility remains incomplete, and knowledge remains fairly limited. Both exogenous exposure and endogenous pathological disorders have been associated with infertility; however, a considerable proportion of infertility cases remain unexplained.

Few studies have directly examined the blood levels of toxic metals in infertile and pregnant women and their relationship to demographic and lifestyle risk factors or hormones. The objective of this study was to examine the blood Pb, Cd, and As levels in infertile and pregnant women. The relationship among blood Pb, Cd, and As levels and reproductive hormone of infertile women was evaluated. We evaluated the association between blood metal levels and relevant variables in infertile women. These variables were assessed using structured questionnaires to collect retrospective data, including relevant demographic, physical, environmental, behavioral, and lifestyle factors. The findings of this study could elucidate the distribution and differences in blood Pb, Cd, and As levels in infertile and pregnant women. Moreover, the results elucidate the association between blood metal levels and the study variables in infertile women.

## Methods

### Study participant recruitment

**Fig. 1:**
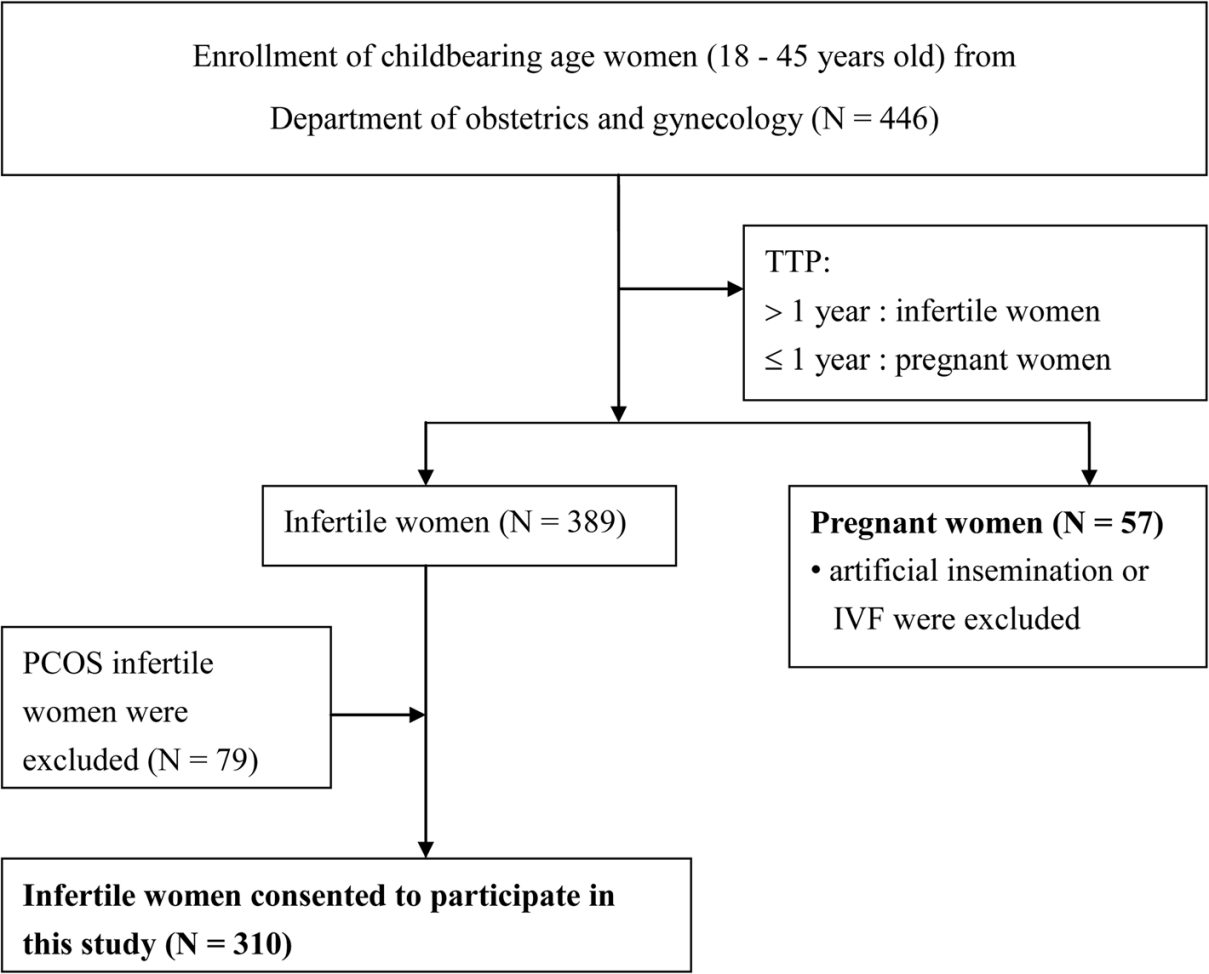
Flow chart of recruiting eligible childbearing age women of this study. TTP: Time to pregnancy. IVF: *In vitro* fertilization

This cross-sectional study investigated women aged 18–45 years. Three hundred and sixty-seven patients treated at the Department of Obstetrics and Gynecology of Taiwan Adventist Hospital between August 2008 and March 2010 were enrolled in this study. Women who routinely took medication or dietary supplements were excluded. We categorized the women as infertile or pregnant according to their reproductive outcomes (Fig. 1).

#### Infertile women

The infertile group comprised women of childbearing age who encountered difficulty conceiving after 1 year of normal sexual activity with the intention to become pregnant [22, 23]. The first exclusion criterion was infertile women with a previous diagnosis of polycystic ovary syndrome (PCOS). Because PCOS is a leading cause of female subfertility [24] and a prevalent endocrine disorder in women, it might interfere with the observation of blood metal and hormone concentrations. The PCOS cases were identified by gynecologists and obstetricians who conducted regular hormone examinations and vaginal ultrasound scans. PCOS diagnosis was made according to the presence of chronic anovulation associated with clinical or biochemical hyperandrogenism. Patients with prediagnosed diabetes mellitus, nonclassical adrenal 21-hydroxylase deficiency, hyperprolactinemia, or androgen-secreting tumors were excluded from the current study [25–27]. Participants diagnosed with congenital adrenal hyperplasia or Cushing syndrome were also excluded. The initial number of infertile women in our study totaled 389. After the exclusion of 79 women with a previous diagnosis of PCOS, our study included a total of 310 infertile women participants.

#### Pregnant women

The pregnant group comprised women of child-bearing age who became pregnant within 1 year of regular unprotected intercourse. Eligible women underwent an ultrasound examination and fetal heartbeat assessment to confirm the pregnancy. For collecting blood samples to represent the exposure of participants before pregnancy, we recruited pregnant women who were in the first 8–10 weeks of gestation. Sixty-five eligible women were initially recruited. Women who received artificial insemination or *in vitro* fertilization (IVF), and women who ultimately did not have a successful delivery were excluded. After excluding five women who had IVF, two women whose pregnancy lasted for more than 10 weeks, and one woman who ultimately did not have a successful delivery, a total of 57 pregnant women were included in this group.

### Data collection

All the participants provided written informed consent before this study began. This study was approved by the Institutional Review Board of Taipei Medical University (approval number: P950045) and the Taiwan Adventist Hospital Investigational Review Board (TAIRB number: 989801A). We collected self-reported participant sociodemographic and lifestyle data for the year prior to study enrollment. Therefore, the lifestyle data, including alcohol consumption and Chinese herbal medicine use, presented the condition of the pregnant participants before pregnancy was confirmed. A trained interviewer administered a standard face-to-face questionnaire to each participant to obtain the potential factors that might reveal their body burden of metals, including sociodemographic information, lifestyle characteristics, anthropometry, and menstruation history [28–30]. Education level was dichotomized as equal to or below senior high school, and university or above. Occupational exposure was confirmed by asking participants whether they had worked in a job that exposed them to toxic metals for at least one month in the year before this study. Average family income was dichotomized as US$3,500 or less per month and greater than US$3,500 per month. Regular menstruation was defined as a 24–35 day a menstruation cycle, and irregular menstruation was defined as menstrual cycle of less than eight times per year or more than 35 days per cycle. Alcohol consumption was defined as drinking in excess of one standard drink (equivalent to 14 g [0.6 ounces] of pure alcohol) per day, based on the recommendation of Dietary Guidelines for Americans (2010) [31]. Participants were classified as smokers if they were current smokers who had smoked more than 100 cigarettes in their entire lifetime [32]. Nonsmokers were defined as women who had never smoked or who had quit smoking for over 1 year before the study [33, 34]. In our study questionnaires, the Chinese herbal medicine items considered were *Angelicae sinensis radix*, *Lycii fructus*, *Zizyphi fructu*, and *Si-Wu-Tang*; these were selected because they are most frequent herbal medicines used by women of childbearing age for achieving optimal health in Chinese society [35].

### Blood sample collection and analysis

For the pregnant women, blood samples were collected within the first 8–10 weeks of gestation. For the infertile women, an overnight fasting blood sample was obtained within the first 3 days of the menstrual cycle for those who ovulated spontaneously, or was obtained randomly from those who had amenorrhea longer than 3 months without hormone-induced withdrawal bleeding.

#### Determination of metal concentrations

Blood samples were collected from each participant in 10 mL ethylenediaminetetraacetic acid tubes. Approximately 1 mL of each blood sample was microwave digested (CEM, Model MDS-2000) with 3 mL of 65 % nitric acid (Suprapur, Merck). Subsequently, we washed the residuals in microwave tubes with 2 % nitric acid and then filtered the digested fluids with 0.45 μm filtered tap water. The total filtered solutions were stored in 15 mL centrifuge tubes. The levels of Pb, Cd, and As were determined using inductively coupled plasma mass spectrometry (ICP-MS; Thermo X-series II). The ICP-MS detection limits for Pb, Cd, and As were 0.23, 0.08, and 0.12 ppb, respectively. The method detection limits for Pb, Cd, and As were 0.74, 0.26, and 0.39 ppb, respectively.

Trace Elements Whole Blood Level 3 (Seronorm™; SERO, Billingstad, Norway) was used as the reference material for the standard material test to ensure the precision and accuracy of the blood metal analysis. The precision levels of Pb, Cd, and As were 97.6, 95.7, and 94.2 %, respectively, and the accuracy values were 100.0, 99.9, and 99.9 %, respectively.

#### Reproductive hormone concentrations in infertile women

Reproductive hormones were measured in whole blood samples that were collected in vacutainers without an anticoagulant, and then centrifuged at 3,000 rpm for 10 min within 2 h after the collection. The samples were then stored at −20 °C until analysis. FSH and LH levels in the test samples were determined using radioimmunoassay kits (Diagnostic Products Corporation, USA and Diagnostic System Inc., USA, respectively) in an accredited laboratory at Taiwan Adventist Hospital (certified by the Department of Health, Taiwan) [36]. The inter-assay coefficients of variation for LH and FSH were 15.1 and 18.3 %, respectively; the intra-assay coefficients of variation for LH and FSH were 6.5 and 8.9 %, respectively. The hormone assay results for the LH and FSH levels are presented as mIU/mL.

### Statistical analysis

The distributions of continuous variables are expressed as mean ± standard deviation (SD). Student’s, Mann–Whitney U, and Kruskal–Wallis tests were conducted to assess the differences in continuous variables between the infertile and pregnant women. A chi-square test was performed to determine the independence of two categorical variables. The Spearman correlation coefficient (ρ value) was used to assess the correlation between blood metal concentrations, hormone levels, and potential variables in the infertile women group. Multiple linear regression was conducted to assess the log transformed blood Pb, Cd, and As levels, and relevant variables in the group. The correlation between blood Pb levels and Chinese herbal medicine use in infertile women was determined using the Spearman correlation test. Adjusted odds ratios (aORs) between relevant variables and elevated blood Pb levels, and their corresponding 95 % confidence intervals (CIs), of infertile women were computed through a logistic regression analysis of blood Pb levels. These blood Pb levels were categorized as higher than the 75th percentile (Q3) or lower than the 25th percentile (Q1), with blood Pb levels in Q1 as the referent. All statistical analyses were performed using SPSS Version 18.0 for Windows (SPSS Inc., Chicago, IL, USA). A 2-tailed test p value of <0.05 was considered statistically significant.

## Results

**Fig. 2:**
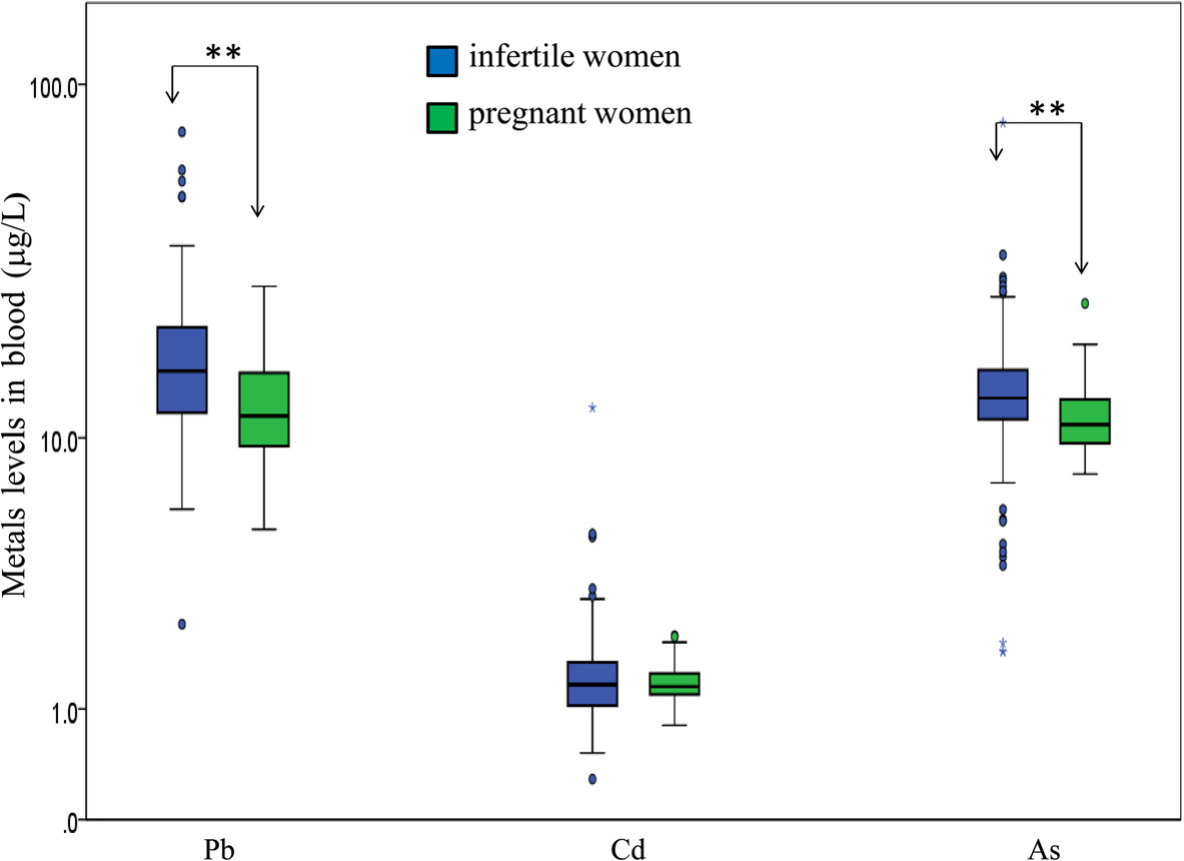
Box and whisker plots display the distribution of the blood Pb, Cd, As levels in participants. Infertile women (*N* = 310) and pregnant women (*N* = 57). ^**^ *p* < 0.01

Table 1 shows a summary of sociodemographic and lifestyle characteristics and the blood metal levels for each research participant. The participants were aged 18–45 years, and the infertile and pregnant women groups exhibited similar mean ages and body mass indices. A significant difference was observed in the categorized frequencies of Chinese herbal medicine use between the groups (*p* < 0.01). In particular, an obvious difference was noted in infertile women taking Chinese herbal medicine 1–6 times per week compared with pregnant women (30.9 % versus 10.5 %). A higher percentage of the infertile women consumed alcohol as usual than did the pregnant women (27.7 % versus 13.2 %, *p* < 0.05). Furthermore, the pregnant women participated in regular physical activity (more than three times per week) more frequently than did the infertile women (32.7 % versus 15.6 %, *p* < 0.05). The blood Pb and As levels were also significantly higher in the infertile women than in the pregnant women (*p* < 0.01). Figure 2 depicts box and whisker plots showing the blood Pb, Cd, and As levels in infertile and pregnant women. The median concentrations of Pb, Cd, and As were 15.7, 1.3, and 13.8 μg/L for the infertile women, and 11.6, 1.3, and 10.9 μg/L for the pregnant women, respectively. The blood Pb and As levels were significantly higher among the infertile women than among the pregnant women (*p* < 0.01).

**Table 1:** Sociodemographic and lifestyle characteristics of participants

Variable	Infertile women (*N* = 310)	Pregnant women (*N* = 57)	p value
	Number (%)	Number (%)	
Age^a^ (years old)	35.2 ± 3.9	34.8 ± 4.1	0.45
Education level			
≤ Senior high school	32(10.3 %)	4(7.1 %)	0.61
University or above	276(89.7 %)	52(92.9 %)	
Occupational exposure^b^			0.81
No	287(92.7 %)	54(94.6 %)	
Yes	23(7.3 %)	3(5.4 %)	
Family income ($/month)			0.41
≤3500	155(50.8 %)	25(43.9 %)	
>3500	150(49.2 %)	32(56.1 %)	
BMI^a^ (kg/m^2^)	21.5 ± 3.5	21.2 ± 2.5	0.62
Menstruation^c^			0.68
Regular	234(78.3 %)	37(82.2 %)	
Irregular	65(21.7 %)	8(17.8 %)	
Fish consumption			0.77
Never	13(4.2 %)	3(5.3 %)	
≤ meal/week	123(39.9 %)	20(35.1 %)	
>1 meal/week	172(55.8 %)	34(59.6 %)	
Chinese herbal medicine use			0.00**
No (<1/month)	140(46.1 %)	39(68.4 %)	
1–3 times/month	71(23.0 %)	12(21.1 %)	
1–6 times/week	94(30.9 %)	6(10.5 %)	
Alcohol consumption			0.04*
No	219(72.3 %)	46(88.5)	
Yes^d^	84(27.7 %)	6(11.5 %)	
Smoking^e^			0.54
No	287(95.0 %)	51(98.1 %)	
Yes	15(5.0 %)	1(1.9 %)	
Physical activity			0.00**
No	90(29.3 %)	5(10.2 %)	
1–2/week	193(62.9 %)	28(57.1 %)	
≥3/week	24(7.8 %)	16(32.7 %)	
Blood metals^a^			
Pb (μg/L)	17.24 ± 8.08	12.56 ± 4.59	0.00**
Cd (μg/L)	1.48 ± 0.89	1.37 ± 0.31	0.79
As (μg/L)	13.90 ± 5.54	11.58 ± 2.92	0.00**

* *p* < 0.05; ** *p* < 0.01

^a^Mean ± SD

^b^Had worked in metal exposure places at least 1 month or not

^c^Menstruation was dichotomized as regular and irregular. Regular: 24–35 day menstrual cycles; irregular: menstrual cycle <8 time/year or >35 days

^d^Drinking wines as usual

^e^No: never-smokers and ex-smokers; Yes: current smokers

In order to interpret the associations on blood Pb levels between infertility and Chinese herbal medicine use. We analyzed the blood Pb levels for infertile and pregnant group by Mann–Whitney *U* test. We dichotomized the variable of Chinese herbal medicine use to “no” and “yes” by the frequency of 1 time/month (Table 2). The results showed that the blood Pb levels of infertile women [14.83 (11.34–18.44) μg/L] were significant greater than that of pregnant women [11.49 (7.78–14.91) μg/L] among no Chinese herbal medicine use (*p* < 0.01). Chinese herbal medicine use may raise the blood Pb levels both in infertile [16.61 (11.94–22.10) μg/L] and pregnant women [14.00 (9.96–17.56) μg/L] (*p* < 0.05).

**Table 2:** Blood Pb levels distribution among women with infertile or pregnancy and dichotomized by Chinese herbal medicine use

Chinese herbal medicine use	Infertile women		Pregnant women		p value
	Median	Q1 - Q3	Median	Q1 - Q3	
No (<1 time/month)	14.83	11.34–18.44	11.49	7.78–14.91	<0.01
	(*n* = 136)		(*n* = 39)		
Yes (≥1 time/month)	16.61	11.94–22.10	14.00	9.96–17.56	<0.05
	(*n* = 163)		(*n* = 18)		

Table 3 shows the correlations among the levels of Pb, Cd, As, and the reproductive hormones in the blood. The blood Cd levels were correlated negatively with blood As levels (*ρ* = −0.12, *p* < 0.05). A significant but weak positive correlation existed between the blood Cd and Pb levels (*ρ* = 0.22, *p* < 0.01), and LH levels were correlated positively with the FSH levels (*ρ* = 0.14, *p* < 0.05).

**Table 3:** Correlations between metals levels and reproductive hormones in blood from infertile women (*N* = 310)

Variable	Pb	Cd	As	FSH
Cd	0.22**			
As	−0.03	−0.12*		
FSH	0.07	−0.01	0.06	
LH	−0.08	0.03	0.00	0.14*

* *p* < 0.05; ** *p* < 0.01

Multiple linear regression models were used to evaluate the influence of study variables (age, educational level, occupational exposure, family income, BMI, menstruation, fish consumption, Chinese herbal medicine use, alcohol consumption, smoking status, and physical activity) on the blood metal levels in the infertile women (Table 4). Chinese herbal medicine use showed a significant correlation to blood Pb levels (*β* = 0.04, *p* = 0.02), menstruation exhibited a significant correlation to blood Cd levels (*β* = −0.06, *p* = 0.05), and family income revealed a significant correlation to blood As levels (*β* = −0.05, *p* = 0.04).

**Table 4:** Multiple linear regression in metals levels and relevant variables of infertile women (*N* = 310)

Variables	Blood Pb		Blood Cd		Blood As	
	β	p value	β	p value	β	p value
Age (years old)	0.00	0.28	0.01	0.14	0.00	0.65
Education level^a^	−0.02	0.69	0.06	0.21	−0.03	0.53
Occupational exposure^b^	0.01	0.79	−0.01	0.85	0.02	0.57
Family income^c^	0.02	0.45	0.01	0.85	−0.05*	0.04*
BMI (kg/m^2^)	0.00	0.96	−0.01	0.24	−0.00	0.74
Menstruation^d^	−0.03	0.31	−0.06	0.05*	−0.03	0.26
Fish consumption^e^	0.01	0.63	−0.02	0.31	0.03	0.09
Chinese herbal medicine use^f^	0.04	0.02*	−0.01	0.49	0.02	0.06
Alcohol consumption^g^	0.00	0.95	0.03	0.29	−0.02	0.50
Smoking^h^	0.11	0.06	−0.06	0.29	0.03	0.51
Physical activity^i^	−0.03	0.24	−0.03	0.12	−0.00	0.91

* *p* < 0.05

^a^Education levels were dichotomized as ≤ Senior high school and University or above

^b^Had worked in metal exposure places at least one month or not

^c^Family income was dichotomized at US$3,500 per month

^d^Menstruation was dichotomized as regular and irregular. Regular: 24–35 day menstrual cycles; irregular: menstrual cycle <8 time/year or >35 days

^e^Fish consumption was categorized in 3 frequencies as never, ≤1 meal/week, and >1 meal/week

^f^Chinese herbal medicine use was categorized in 3 frequencies as no (<1/month), 1–3 times/month, and 1–6 times/week

^g^Alcohol consumption was dichotomized as yes and no. Yes defined as drinking wines as usual

^h^Smoking was dichotomized as yes and no. Yes: current smokers; No: never-smokers and ex-smokers

^i^Physical activity was categorized in 3 frequencies as no, 1-2/week, and ≥3/week

We further analyzed relevant variables and the blood Pb levels in the infertile women by using a logistic regression model adjusted for potentially relevant predictors. We initially tried to assess the dose-response effect for quartile of blood Pb. Compared to the referent blood Pb levels lower than Q1, the dose-response effect could not be observed (data not shown). Nevertheless, compared with the referent blood Pb levels lower than Q1, elevated blood Pb levels (higher than Q3) were found in categorized group of Chinese herbal medicines use 1–6 times per week (aOR = 2.82, 95 % CI 0.98–8.09). The high Pb burden might result from consuming Chinese herbal medicines, even though marginal significance (*p* = 0.05). Exercising 1–2 times per week was also marginally significant (*p* = 0.05); therefore, exercise might reduce Pb accumulation in infertile women (aOR = 0.37, 95 % CI 0.14–1.00). Finally, we presented the results compared with the referent blood Pb levels lower than Q1, and blood Pb levels higher than Q3 in the Table 5.

**Table 5:** Adjusted^a^ logistic regression analyses in relevant variables and blood Pb levels^b^ of infertile women

Variables	aOR	95 % CI	p value
Age (years old)	1.07	0.96–1.19	0.20
Occupational exposure^c^			
No	1		
Yes	0.83	0.15–4.51	0.83
Family income ($/month)			
≤3500	1		
>3500	1.17	0.48–2.82	0.73
Menstruation^d^			
Regular	1		
Irregular	1.43	0.49–4.14	0.51
Chinese herbal medicine use			
No (<1/month)	1		
1–3 times/month	0.90	0.32–2.52	0.84
1–6 times/week	2.82	0.98–8.09	0.05
Alcohol consumption^e^			
No	1		
Yes	0.90	0.36–2.27	0.83
Physical activity			
No	1		
1–2/week	0.37	0.14–1.00	0.05
≥3/week	0.38	0.11–1.29	0.12

^a^Adjusted on age, occupational exposure, family income, menstruation, Chinese herbal medicine use, alcohol consumption, physical activity

^b^Blood Pb levels in the regression was by evaluated blood Pb levels ≥ Q3(75th) (21.00 μg/L) compared to the referent; the blood Pb levels ≤ Q1 (25th) (11.83 μg/L) is as the referent

^c^Had worked in metal exposure places at least one month or not

^d^ Menstruation was dichotomized as regular and irregular. Regular: 24–35 day menstrual cycles; irregular: menstrual cycle <8 time/year or >35 days

^e^Alcohol consumption was dichotomized as yes and no. Yes defined as drinking wines as usual

**Fig. 3:**
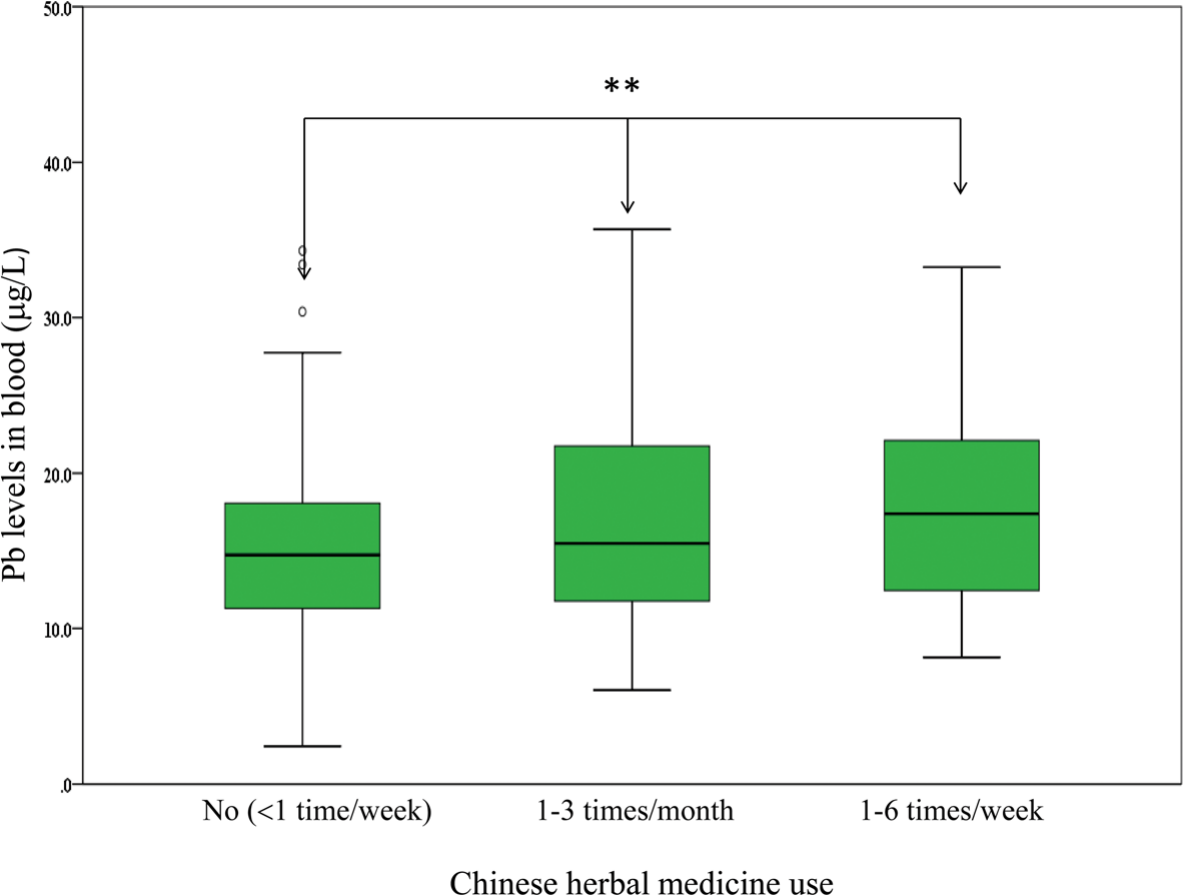
Blood Pb levels in infertile women by 3 categorized frequencies of Chinese herbal medicine use. ** *p* < 0.01

As shown in Fig. 3, blood Pb levels increased with the frequency of Chinese herbal medicine consumption among the infertile women. Chinese herbal medicine consumption was classified into three frequencies: none, 1–3 times per month, and 1–6 times per week. Blood Pb concentration was observed to increase with frequency of Chinese herbal medicine consumption. The geometric mean blood Pb levels in the none, 1–3 times per month, and 1–6 times per week groups were 14.46, 15.67, and 16.95 μg/L, respectively (Fig. 3, *p* < 0.01).

## Discussion

This is one of the few studies assessing the blood levels of Pb, Cd, and As, along with other relevant variables, by categorizing women of childbearing age as infertile or pregnant. Our results indicate that blood Pb and As, but not Cd, levels were significantly higher in the infertile women compared with the pregnant women. In addition to the high blood metal levels, we observed some lifestyle differences in the infertile women, including greater consumption of alcohol and Chinese herbal medicine and a lack of physical activity.

Bloom et al. [3] reported that the respective blood Pb levels in pregnant and nonpregnant women were 15.44 ± 1.23 and 15.54 ± 1.57 μg/L, respectively; this is comparable to our findings. According to the nationwide Environmental Health Monitoring System in the Czech Republic, the median blood Pb levels of 18- to 58-year-old women was 14 μg/L in 2009 [37], which is also comparable to our results. Furthermore, Chang et al. [21] investigated the relationship between Pb exposure and the risk of infertility in women between 2000 and 2001 in Taiwan, reporting mean blood Pb levels of 35.5 and 27.8 μg/L in infertile women and pregnant women, respectively. The blood Pb levels reported in that study are significantly higher than those observed in our study, possibly because lead fuels were prohibited in Taiwan in 2000.

Bloom et al. [3] studied 18- to 34-year-old women and reported blood Cd levels of 1.67 ± 2.82 and 1.52 ± 0.95 μg/L in pregnant and nonpregnant women, respectively, the results were similar to our study. Recent data from Ikeda et al. [38] reported that the geometric mean of Cd concentrations in the blood of women in nonpolluted areas of Japan was 1.23 μg/L. Additionally, a 2008–2010 national surveillance study in South Korea revealed that the geometric mean Cd blood concentrations was 1.20 μg/L in 40- to 49-year-old women [39]. These findings are also comparable to our results. Among nonoccupationally exposed people, tobacco is the leading source of exposure to Cd [40, 41]. However, in this study, a nonsignificant difference in blood Cd levels was observed between the infertile and pregnant women (Table 1), potentially because of the low percentage of participants who were smokers (5.0 % of infertile women and 1.9 % of pregnant women). Compared with the results of our study and previous findings [3, 42, 43], a variety range of blood As levels in women were observed.

Many infertile women use Chinese herbal medicine to assist them in conceiving, but doing so may expose them to Pb, Cd, and As. According to a report from the Division of Research and Analysis of the Taiwan Food and Drug Administration [44], these toxic metals can be found in ten common raw Chinese herbs. The 80th percentile of each heavy metal in these unprocessed herbs is within 0.19–6.53 mg/L for Pb, 0.04–1.68 mg/L for Cd, and 0.09–2.34 mg/L for As. Toxic metals such as Pb, Cd, and As, which have been detected in commercially available Chinese herbal medicines, may pose a health risk to these women [35, 45, 46]. *Si-Wu-Tang* is also used to treat irregular menses, period pain, overactive fetuses, anemia, and other blood stasis conditions. *Si-Wu-Tang* has the highest lead concentration among all Chinese herbal products, with a level approximately 8.8–16.6 times higher than other herbs [35]. Wu et al. [47] urged greater attention be paid to high Pb exposure from Chinese herbal medicine consumption. The infertile women in the Wu et al. study were more likely to consume Chinese herbal medicine than were the pregnant women. These findings were consistent with our study results of Table 2 that Chinese herbal medicine use might be one of important source of Pb exposure in childbearing age women, and the elevated blood Pb level might be the risk factor that causes women infertility. The correlation between Chinese herbal medicine use and blood As levels was borderline significant in infertile women (Table 4; *p* = 0.06), which might indicate a certain contribution to greater blood As levels from Chinese herbal medicine use. Among the infertile women, blood As levels were significantly higher among those in the lower median family income group. This result was similar to those of other studies that reported a significant negative correlation between income and As exposure [48, 49].

Anderson et al. [50] suggested that maintaining a healthy lifestyle could assist women in conceiving, and that, to aid in conception, infertile couples should adopt a healthy lifestyle that includes regular physical activity, not smoking, and not consuming alcohol. According to previous studies, the perspiration caused by increased physical activity can increase heavy-metal excretion [51–53], which may reduce blood Pb, Cd, and As levels and enhance fecundity. As shown in Table 1, the infertile women habitually consumed alcohol, markedly higher (27.7 %) than that of pregnant women (11.5 %). Alcohol can have direct adverse effects on the maturation of the ovum [54, 55], ovulation, early blastocyst development, and implantation [56]. Several studies have observed an alcohol-induced increase in estrogen levels in animals and healthy women [57, 58], which may reduce FSH secretion, suppress folliculogenesis, and further affect ovulation. These causes may result in adverse fecundity among women of reproductive age. From our study results of Table 1, a greater percentage of alcohol consumption as usual in infertile women than that in pregnant women (27.7 % versus 11.5 %, *p* < 0.05), and regular physical activity more frequently in the pregnant women than that in infertile women (32.7 % versus 15.6 %, *p* < 0.05). These results would indicate that women might conceive difficutly due to risk behaviors.

The relationships among toxic metals in the blood have been examined in previous studies. One study found that a significant correlation between the presence of Pb and Cd in cattle [59]. A study examining Pb-exposed and nonexposed workers found interactions between blood Cd and As levels [60]. Significant correlations between blood Pb and Cd levels and Cd and As levels were observed in our study (Table 2), but the causes and mechanisms of these relationships require further study. Pb, Cd, and As could be endocrine-disrupting chemicals [61] and could interact with hormones and disrupt endocrine functions that might exert reproductive problems in female [62]. This study yielded no significant correlations between blood metals and hormone levels, which may have occurred because most of the infertile participants lacked occupational or environmental exposure. Although blood Pb levels were significantly higher in the infertile women than in the pregnant women, the Pb exposure levels of these populations were below hazard reference values. The median blood Pb concentration was 15.7 μg/L in the infertile women of our study; yet the U.S. Centers for Disease Control and Prevention recommends that blood Pb levels not exceed 100 μg/L in women to protect their offspring from toxic effects [63]. This high threshold may be why no obvious effect on reproductive hormones was revealed.

The strength of this study is its assessment of the distribution and differences of the blood metal levels of infertile and pregnant women. However, some bias existed in this study, which is a common limitation of retrospective investigations [64, 65]. Another limitation of this study is that some data regarding sociodemographic and lifestyle factors were self-reported. No dietary record and consumption duration (Chinese herbal medicine) were included in our questionnaire, which may have led to the underestimation of participants’ exposure to toxic metals. The potential exposure levels of toxic metals were not collected from the residential areas of our participants; this may also have affected their actual blood metal levels. Certain clinical conditions such as malabsorption may also induce an underestimation of toxic metal concentrations in the blood. Additionally, recruiting healthy pregnant women for this research was difficult because they may have less motivation to participate in medical studies; the number of pregnant women in our study (*N* = 57) was lower than that of the infertile group (*N* = 310). However, we conducted a power of significance test for blood Pb levels in the pregnant and infertile women, and *p* > 0.9. Therefore, even though fewer pregnant women were recruited, the sample was large enough for comparison with the infertile group. Another limitation of this study is that essential metals such as iron, selenium, zinc, magnesium, and calcium, were not measured. These metals may interfere with reproduction in women [2, 66, 67]. More than 80 % of the infertile women in our study came from urban areas. The average family income in Taiwan was estimated to be US$3,000 per month according to a Taiwan government report [68], and family income was more than US$3,500 per month for approximately 50 % of participants in our study. For these reasons, we could assume that the infertile women in this study received adequate nutrition and had a relatively higher socioeconomic status, but no assessment was conducted to determine whether they had any deficiency in essential metals. Moreover, the interaction of essential and toxic metals on hormone levels was not investigated in this study; thus, additional research is necessary to determine whether this interaction affects hormones levels or produces any distinct effects in infertile women.

## Conclusion

Among the variables examined in this study, alcohol consumption, Chinese herbal medicine use, and a lack of exercise was more common among the infertile women than among the pregnant women. Frequently consuming Chinese herbal medicine may have caused elevated blood Pb levels, and physical activity may have reduced the accumulation of Pb among the infertile women. The caution of Chinese herbs use is warranted to prevent toxic metals from accumulating in the blood. Regular physical activity may reduce the accumulation of Pb in the body and improve women’s health.

## Abbreviations

Pb: Lead
Cd: Cadmium
As: Arsenic
FSH: Follicle-stimulating hormone
LH: Luteinizing hormone
PCOS: Polycystic ovary syndrome
IVF: *In vitro* fertilization
SD: Standard deviation
aOR: Adjusted odds ratio
CI: Confidence interval
Q1: the 25th percentile
Q3: the 75th percentile
